# The role of mesenchymal stem cell-derived exosome in epigenetic modifications in inflammatory diseases

**DOI:** 10.3389/fimmu.2023.1166536

**Published:** 2023-05-16

**Authors:** Zihan Zhao, Li Zhang, Dickson Kofi Wiredu Ocansey, Bo Wang, Fei Mao

**Affiliations:** ^1^ Key Laboratory of Medical Science and Laboratory Medicine of Jiangsu Province, School of Medicine, Jiangsu University, Zhenjiang, China; ^2^ Nanjing Lishui People’s Hospital, Zhongda Hospital Lishui Branch, Southeast University, Nanjing, China; ^3^ Directorate of University Health Services, University of Cape Coast, Cape Coast, Ghana

**Keywords:** epigenetic modification, inflammatory bowel disease, osteoarthritis, systemic lupus erythematosus, mesenchymal stem cell-derived exosome

## Abstract

Epigenetic modification is a complex process of reversible and heritable alterations in gene function, and the combination of epigenetic and metabolic alterations is recognized as an important causative factor in diseases such as inflammatory bowel disease (IBD), osteoarthritis (OA), systemic lupus erythematosus (SLE), and even tumors. Mesenchymal stem cell (MSC) and MSC-derived exosome (MSC-EXO) are widely studied in the treatment of inflammatory diseases, where they appear to be promising therapeutic agents, partly through the potent regulation of epigenetic modifications such as DNA methylation, acetylation, phosphorylation, and expression of regulatory non-coding RNAs, which affects the occurrence and development of inflammatory diseases. In this review, we summarize the current research on the role of MSC-EXO in inflammatory diseases through their modulation of epigenetic modifications and discuss its potential application in the treatment of inflammatory diseases.

## 1 Introduction

MSCs are widely used in the field of regenerative medicine and have the potential to differentiate into adipose, bone, cartilage, and other tissues under specific *in vitro* conditions. Classical MSCs express CD105, CD73, CD90, CD34, and CD44 and these surface markers are commonly used in research to identify the purity of MSCs by flow cytometry (1). There are many sources of MSCs, mainly bone marrow, adipose, and umbilical cord blood (2). Currently, MSCs are widely being explored in the treatment of autoimmune diseases, inflammatory diseases, and many other diseases (3). MSCs secrete exosomes, a type of extracellular vesicle (EV) with a diameter of 40-200 nm, and are the best-defined secretory vesicle of all EVs. Exosomes are produced when the endosomal membrane invaginates to form multi-vesicular bodies (MVBs) and are released when the MVBs fuse. The exosome membrane is structurally similar to the cell membrane, rich in signaling molecules and surface antigens, and it also contains a typical lipid raft structure. The main mechanisms by which exosomes bind to target cells include ligand-receptor binding, endocytosis, and direct binding (4). MSC-EXO has similar functions to MSCs as recent evidence suggests that MSCs act primarily through paracrine effects and the use of MSC-EXO as a vehicle for cell-free therapy reduces the concern of injecting live cells (5, 6). Therefore, it has been suggested that MSC-EXO is superior to MSCs in clinical treatment, and how to apply MSC-EXO in the clinical setting is a trending topic of research today (7).

Epigenetics has been a headline of research in various fields worldwide, and the study of epigenetic modifications has contributed to the development of medicine, biology, and other disciplines. It has also led to breakthroughs in the study of disease pathogenesis, diagnosis, and treatment. The study of epigenetics includes two main aspects; the regulation of selective transcriptional expression of genes and the post-transcriptional regulation of genes. From a broader perspective, epigenetics is the study of the mechanisms by which gene expression is regulated and, the mechanisms by which genes interact with each other. The core of epigenetic research and the key question to be addressed is the central law of regulation of the transmission of genetic information from the genome to the transcriptome (8). Epigenetic modifications include histone-related modifications such as histone methylation, acetylation phosphorylation, DNA methylation, and expression of regulatory non-coding RNAs (9, 10). Epigenetic modifications are dynamic and can be caused by genetic factors or induced by environmental factors. Studies have shown that epigenetic modifications are closely associated with the development and progression of many diseases. The interplay and intermodulation between epigenetic remodeling and metabolic reorganization are thought to be one of the hallmarks of inflammatory diseases, Various metabolic and epigenetic changes, to a certain extent, promote the development of inflammation, leading to the onset of chronic inflammatory diseases (11). For example, fibrosis, a consequence of chronic inflammatory disease, is caused by a variety of factors including epigenesis. The expression of DNA methyltransferase (DNMT) in patients with idiopathic pulmonary fibrosis (ITP) is significantly higher than that in normal people, indicating that the abnormal expression of DNMT is one of the important factors leading to the generation of ITP (12). In addition, the degree of DNA methylation of peroxisome proliferator-activated receptor γ in plasma is one of the important indicators used to assess the severity of pulmonary fibrosis clinically (13). A number of drugs that use modulation of epigenetic modifications to treat diseases are in development or already in clinical use, and drugs that modulate DNA methylation and histone modifications are already widely used in the treatment of diseases such as cancer (**Table 1**).

**Table 1:** Drugs used in clinical practice for cancer and inflammatory disease.

Modifying objects	Mechanism	Drugs	Disease	Reference
DNA	DNA methylation	Azacytidine decitabine EGCG Clofarabine	Leukemia	(14, 15, 113)
Histones	Acetylation, methylation, ubiquitination, benzoylation, deimination, and poly(ADP) ribosylation	Panobinostat tazemetostat Pracinostat Vorinostat	MM refractory B cell lymphoma AML CTCL	(16, 17)
RNA	RNA interference	Alicafforsen AZD8601	IBD Ischemic heart disease	(18, 19)

Inflammation is a biological response that occurs when the body is stimulated by pathogens, microorganisms, damaged cells, and other internal and external environmental factors. Under normal physiological conditions, inflammation activates the body’s immune system to eliminate the corresponding stimulus, initiates the healing process, minimizes tissue damage and infection caused by the stimulus, helps restore homeostasis, and is an important defense mechanism in maintaining host health. However, prolonged failure of inflammation to subside can result in chronic inflammation and tissue damage, which contributes to the pathogenesis of many inflammatory diseases such as atherosclerosis, IBD, and OA (10). Inflammatory diseases are characterized by a dramatic increase in the expression of pro-inflammatory cytokines (20, 21) such as Interleukin-6 (IL-6) and Interleukin-β (IL-β) and activation of the NOD-like receptor thermal protein domain associated protein 3 (NLRP3)inflammasome. Inflammatory diseases are often difficult to treat clinically, difficult to cure, and prone to recurrence (22). Therefore, there is an urgent need to explore new and effective therapeutic approaches for inflammatory diseases. This review summarizes previous studies on epigenetic abnormalities in inflammatory diseases and how MSC-EXO affects the occurrence and development of inflammatory diseases by influencing epigenetic modification.

## 2 Epigenetic modifications

Epigenetic modification is a complex physiological mechanism that is important for maintaining homeostasis. It regulates gene expression without changing the DNA sequence. Epigenetic changes are dynamic, and influenced by age, environment, and many other factors (23). Epigenetics includes DNA methylation, histone modification, and RNA-based mechanism. DNA methylation is the transfer of a methyl group from the methyl donor to the fifth carbon of DNA cytosine residue to form a specific methylation structure(5-MC), occurring mostly on CPG islands in the gene promoter region, and methylation in gene promoter region can lead to transcriptional silence. This process is catalyzed by the DNA methyltransferase family (DNMT). Different DNMTs play different roles in DNA methylation (24). Abnormal DNA methylation is closely related to a variety of diseases. For example, abnormal hypermethylation is the most important epigenetic modification mechanism for atherosclerosis (25). Overexpression of DNMT1 and DNMT3A can lead to abnormal methylation of DNA of tumor suppressor genes (TSGs), leading to pituitary adenoma invasion. Therefore, DNA methylation is closely related to the occurrence and development of invasive pituitary adenoma (26). DNA methylation-based biomarkers and epigenetic therapy play an important role in the early diagnosis and prognosis of a variety of diseases.

A nucleosome consists of DNA and a histone octamer and the N-terminus and C-terminus of histones can be modified by posttranslational modification such as the addition of methyl groups to lysine residues of histones H3 and H4 to induce methylation of histones or phosphorylation of histones at serine and tyrosine residues. In addition to this, there are acetylation and ubiquitination as types of histone modifications (27). Histone modification is catalyzed by a variety of enzymes called histone acetyltransferases (HATs) and histone deacetylases (HDACs), regulating histone acetylation. Histone methyltransferase (HMT) catalyzes the methylation of histones by transferring methyl groups from S-adenosyl methionine (SAM) to lysine residues of histones (28). Histone modification has been shown to play a role in a variety of diseases such as non-small lung cell carcinoma (NSCLC), periodontitis, and breast cancer (29, 30). Di Zhang et al. reports a novel post-translational modification of proteins, histone lactate modification. Their study showed that the accumulated lactic acid in the body can catalyze the lactating modification of histone lysine to further regulate gene expression (31), and found that histone lactating modification plays an important role in the regulation of inflammation and tumor metabolism. Histone lactate modification in the tumor microenvironment can generate immunosuppression to promote the immune escape of tumors (32). Histone lactate modification also improves cardiac function after myocardial infarction by promoting repair and gene transcription to regulate the dual activities of anti-inflammation and angiogenesis by monocyte-macrophage (33).

**Figure 1:**
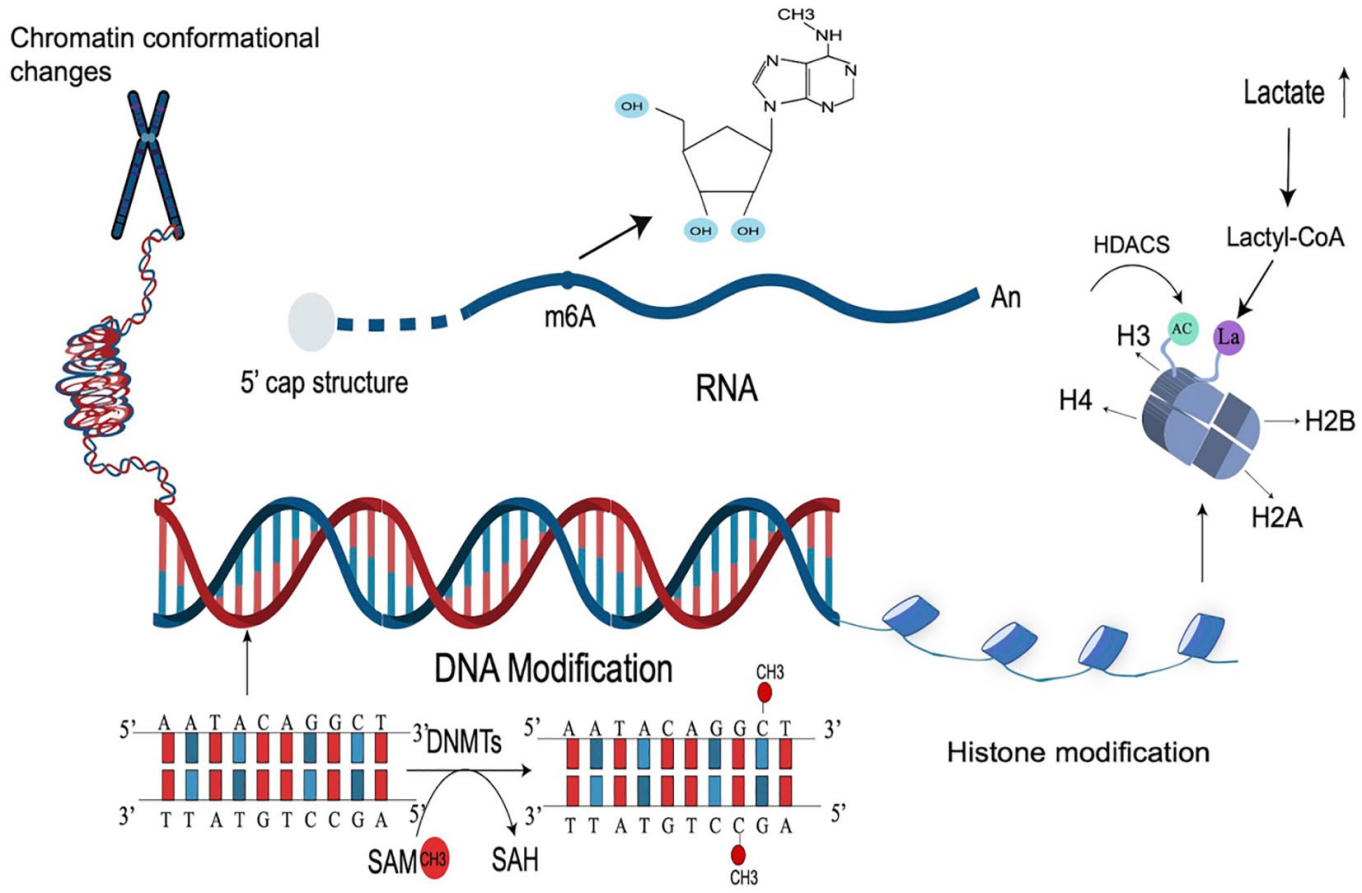
Types of epigenetic modifications. Epigenetic modification is a very complex mechanism that regulates gene expression, mainly including DNA modification, histone, and RNA modification, as well as chromosome changes. The most common epigenetic modification is DNA methylation (catalyzed by DNMTs), RNA m6A modification, and histone acetylation modification (catalyzed by HDACs). Lactate-induced histone modification is a newly discovered histone modification.

RNA modification is also an important part of epigenetic modification (34). Similar to DNA modification, cellular RNA also has a variety of chemical modifications, such as N6-methylladenosine (m6A) of mRNA. m6A modification is the most abundant epigenetic modification of RNA and is mainly catalyzed by the methyltransferase complex, which is composed of METTL3, METTL4, and other protein subunits. Abnormal modification of m6A leads to abnormal transcription, resulting in an abnormal translation program, and promotes the occurrence and development of tumors. Studies have shown that m6A modification can also affect the occurrence and development of IBD by regulating immune cells and RNA. YTH domain family 2 (YTHDF2) is an m6A-binding protein and its knockout increases inflammation. The expression of YTHDF2 is closely related to the development of IBD (35, 36). The chemical modification of these RNAs plays a very important role in RNA metabolism. The types of epigenetic modification are briefly shown in **Figure 1**.

## 3 The effect of MSCs and MSC-EXO on epigenetic modifications in inflammatory diseases

**Figure 2:**
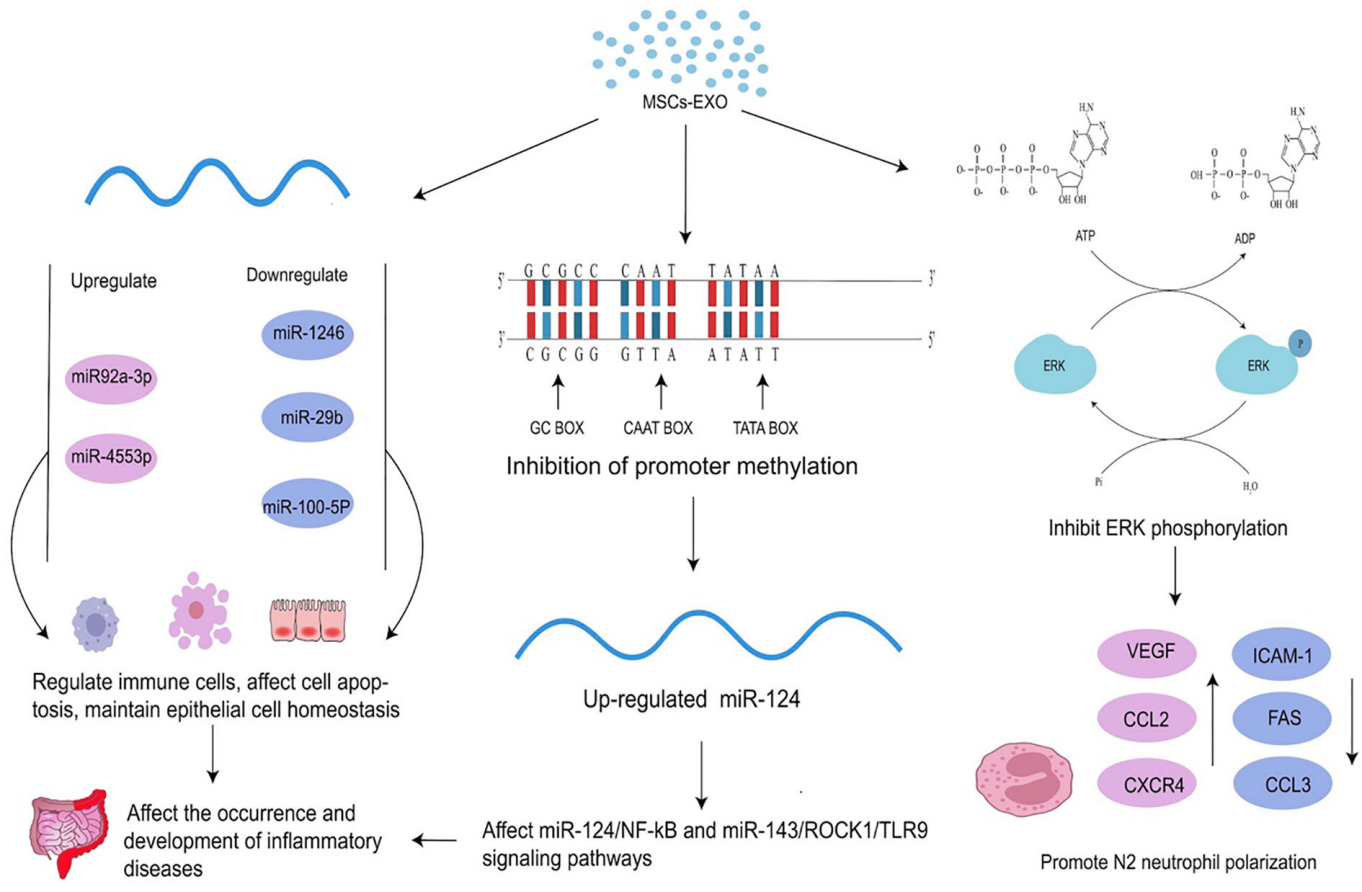
Effects of MSC-EXO on epigenetics. MSC-EXO can affect mRNA expression by inhibiting promoter methylation and regulating the expression of related molecules to affect the occurrence and development of diseases. It can also directly regulate the expression of mRNA and other RNA. MSC-EXO can ameliorate the occurrence and development of inflammatory diseases by influencing enzyme activity to up-regulate or down-regulate ERK phosphorylation and promote N2 neutrophil polarization.

The relationship between epigenetic modifications and MSCs is one of mutual influence and regulation. Epigenetic modifications play an important role in the differentiation of MSCs as DNA methylation, histone modifications, and miRNAs are all important mechanisms that regulate MSC differentiation (37). MSC-EXO can also mitigate the onset and progression of inflammatory diseases by regulating the epigenetic modifications of related molecules. Changes in epigenetic modification are common in inflammatory diseases (**Table 2**). MSC-EXO can influence the expression of epigenetic modifications related molecules by regulating DNA methylation levels to suppress inflammation *in vivo*, leading to the amelioration of inflammatory diseases (55). It can also be used to activate relevant signaling pathways through RNA modifications such as mRNA m6A modifications to suppress inflammation and restore homeostasis in the body’s internal environment (56). Moreover, MSCs play an immunomodulatory and homeostatic role in inflammation, and MSCs-EXO can contribute to the resolution of inflammatory diseases by regulating the polarization of macrophages from pro-inflammatory M1 to anti-inflammatory M2 (57) and inducing cytokine secretion to participate in the immunomodulatory of T cells (58) (**Figure 2**).

**Table 2:** Abnormal epigenetic modification common in inflammatory diseases.

Disease	Epigenetic modification	Therapeutic target	Key outcome	Reference
Atherosclerosis	Abnormal DNA methylation mediated by DNMT1 and DNMT3a	DNA methylation inhibitors (5-azil and its nucleoside analogues)	In mice treated with DNA methylation transferase inhibitors, macrophage activation was inhibited and macrophage invasion of atherosclerotic plaques was reduced	(38, 39)
Atherosclerosis	Loss of HDAC3 results in abnormal acetylation of histones	HDAC3 inhibitors	HDAC3 inhibitor suppresses EndMT *via* modulating inflammatory response in ApoE-/- mice and HUVECs.	(40, 41)
Atherosclerosis	Increased trimethylation of histone H3K27	Histone methylation inhibitors (e.g., EZH2 inhibitors)	Mice treated with EZH2 inhibitor GSK126 showed significant reductions in atherosclerotic plaque	(42, 43)
Inflammatory bowel disease	Abnormal modification of mRNA m6A	m6A eraser	FTO protects IBD patients from adverse effects after treatment with thiazolate	(44, 45)
Inflammatory bowel disease	Abnormal DNA methylation	DNA methylation inhibitors	Several immuno-active genes show significant correlations between methylation and gene expression in IBD, and multi-omic integration of the methylome, genome, and transcriptome also implicate specific pathways in immune activation, response, and regulation at disease inception.	(46)
Inflammatory bowel disease	Abnormal histone acetylation levels	HDAC inhibitor	In mice treated with VPA, acetylation of histone 3 at the inflammatory site increased, alleviating DSS-induced colitis	(47, 48)
Osteoarthritis	Abnormal DNA methylation	DNA methylation inhibitors	Compared with normal cartilage, OA has a reduced methylation signature, which is associated with increased gene expression and leads to ECM degradation	(49)
Osteoarthritis	promoter methylation of BPM-7	methylation inhibitors	Altered expression levels of BMP-7 may lead to abnormal matrix synthesis and cartilage degeneration	(50, 51)
Systemic lupus erythematosus	DNA hypomethylation in T cells	DNA methylation inhibitors	CD4+ T cells convert to autoreactivity when treated with 5-azacytidine, procainamide or hydralazine, all of which inhibit DNA methylation and can lead to a lupus-like syndrome	(52, 53)
Systemic lupus erythematosus	Low acetylation of histones H3 and H4 in CD4+ T cells	Immunomodulator	Mycophenolic acid (MPA) can up-regulate the global acetylation status of histone H3/H4 by regulating HAT and HDAC in CD4(+)T cells in lupus.	(54)

### 3.1 Inflammatory bowel disease

Inflammatory bowel disease (IBD) is a chronic, idiopathic, non-specific inflammatory disease of the gastrointestinal tract, mainly divided into two categories: UC and CD. The specific pathogenesis of IBD is not yet clear but a combination of genetic, immunological, and microbial factors certainly contribute to its onset and progression (59, 60). The main clinical manifestations of IBD are gastrointestinal symptoms such as abdominal pain, diarrhea, and bloody stools, with some patients presenting with extra-intestinal symptoms (EIM). EIM most often affects the joints, skin, or eyes, causing peripheral arthritis and erythema nodosum, but can also affect other organs such as the liver and pancreas (61). There are various clinical strategies for the treatment of IBD such as the use of traditional methods like immunomodulators and the ongoing exploration of fecal microbiota transplantation (15). The incidence of IBD in Western countries is entering the Compounding Prevalence stage, heading towards an eventual Prevalence Equilibrium stage, whereas, in some Asian countries, the incidence is steadily increasing as technology and living standards increase. Thus, there is an urgent need to explore the pathogenesis of IBD and new effective treatments (62, 63).

Epigenetic alterations are closely related to the development and progression of IBD. Gloria et al. found that the rate of 3H-methyl group admixture in the DNA of UC patients was 10 times higher than normal and that patients with the histologically active disease also had significantly higher rates, demonstrating that DNA methylation is associated with the pathogenesis of UC. Histone deacetylase (HDAC) inhibitor can promote acetylation of intestinal epithelial cells and the deficiency of HDAC1 exacerbates DSS-induced colitis in a mouse model of IBD (64, 65). Thus, repairing abnormal epigenetic expression in patients with IBD may also be one of the possible therapies for IBD, and controlling or reversing abnormal epigenetic modifications may provide some relief from the symptoms of IBD.

Studies have shown that MSCs can alleviate IBD by modulating epigenetic modifications of related molecules, Yi-Jun et al. found that enterocytes that were co-cultured with Methyltransferase-like 3 (METTL3) and Insulin-like growth factor 2 mRNA binding protein 3 (IGF2BP3) overexpressed or decreased MSC-EXO. Methylated RNA immunoprecipitation-quantitative polymerase chain reaction (MeRIP-qPCR) showed that the level of m6A modification of pre-miR-34A was significantly increased in the METTL3 overexpression group and promoted a marked increase in the expression of miR-34a-5p, which upregulated the tight junction proteins ZO-1, Occludin, Zonulin, and Claudin. Tight junction proteins play a critical role in maintaining intestinal function and the integrity of the intestinal barrier (66, 67), and miR-34a-5p is closely associated with anti-apoptosis (68). TUNEL and TER results showed that the apoptosis of intestinal epithelial cells in IBD mice was decreased. In contrast, the deletion of METTL3 and IGF2BP3 resulted in a significant decrease in the level of m6A modification of pre-miR-34A, which in turn led to reduced levels of miR-34a-5p and increased apoptosis. The same results were shown in animal experiments, where mice that received tail vein injections of MSCs that overexpressed METTL3 and IGF2BP3 showed reduced damage to the colorectal mucosa and increased levels of miR-34a-5p. In contrast, mice that received tail vein injection of METTL3 or IGF2BP3 knockout MSCs showed no alleviation or even increase in colorectal damage, and the level of miR-34a-5p was reduced. This suggests that MSCs can modulate the level of pre-miR-34a m6A modification through METTL3/IGF2BP3 to improve the stability of pre-miR-34a, promote miR-34a-5p secretion, maintain intestinal function, and protect the integrity of the intestinal barrier Maintaining the integrity of the intestinal barrier and intestinal function is also very important in the treatment of IBD, and MSCs play a therapeutic role in IBD by regulating the epigenetic modification of pre-miR-34A through METTL3 and IGF2BP3 (69).

**Figure 3:**
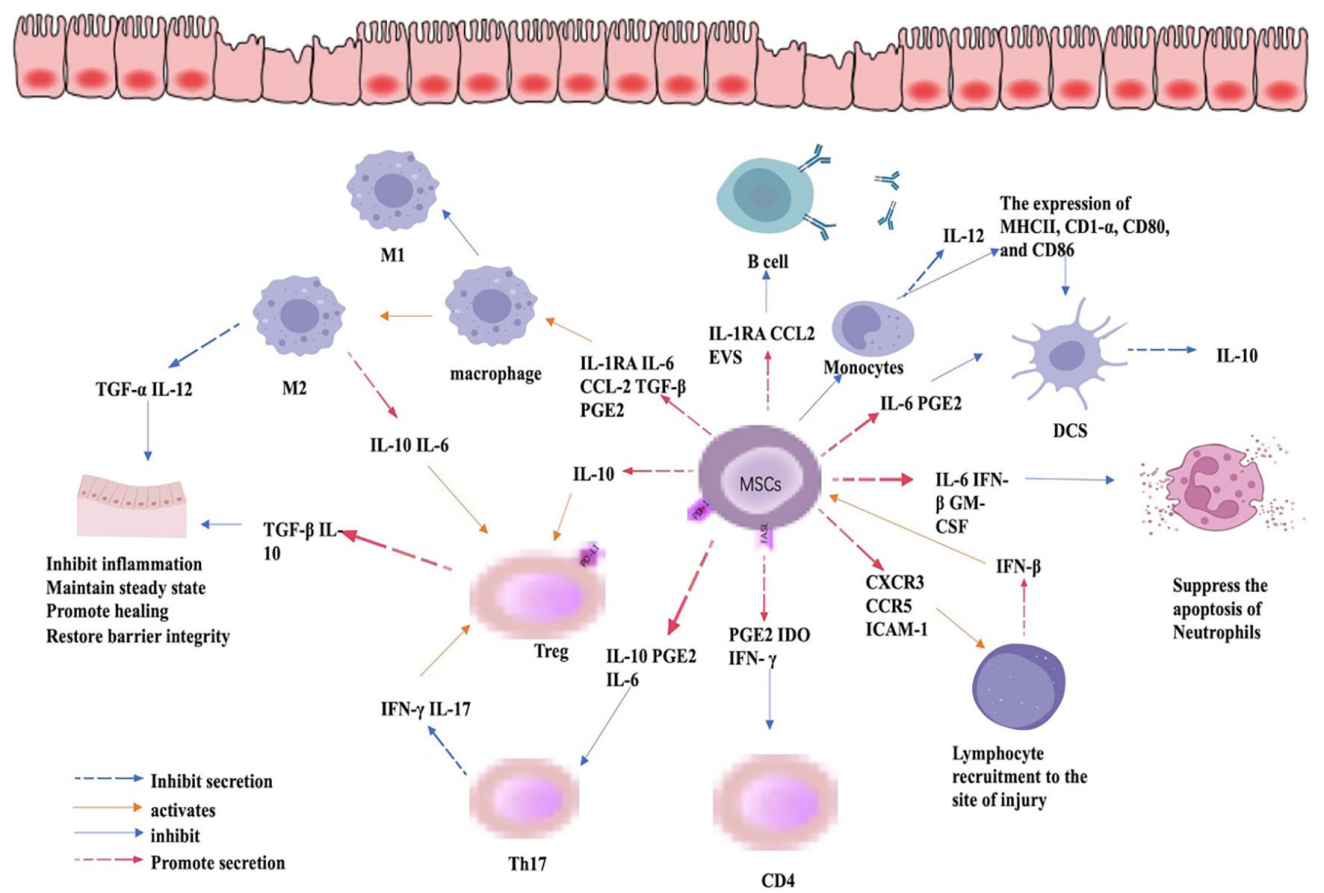
Immunomodulatory role of MSC in IBD. By regulating the expression of cytokines, MSCs can inhibit the expression of CD4 and Th17cells, promote the differentiation of Treg cells, the differentiation of macrophages to M2 phenotype, and inhibit the apoptosis of neutrophils and the expression of pro-inflammatory cytokines, thus mitigating inflammation, promoting mucosal healing, and restoring barrier function.

MSC-EXO has also been widely used in the study of IBD, as it regulates the immune response by inducing Treg cells and tumor necrosis factor-α stimulated gene 6 (TSG-6) to repair the intestinal barrier, relieving the occurrence and progression of IBD (70, 71). In addition to the above mechanisms, MSC-EXO, like MSC, can also mitigate IBD by regulating epigenetic modifications. Huashan Liu et al. found that MSCs-EXO can reduce the level of phosphorylated IκBα in macrophages, reduce the nuclear translocation of NF-κB p65 subunit, increase the level of total IκBα protein and mRNA, thus inhibiting inflammation and alleviating IBD. MSC-EXO-transmitted metallothionein-2 to inhibit NF-κB activation by promoting IκBα transcription and inhibiting IκBα phosphorylation, thereby further abrogating inflammation (70). The study of Gaoying Wang and colleagues showed that human umbilical cord MSC-EXO (hUCMSC-EXO) effectively alleviates the symptoms of IBD induced by DSS in mice, where the number of neutrophils in the gut of hUCMSC-EXO treated mice was reduced along with inhibited ERK phosphorylation and increased N2-phenotype. Clinical studies show that patients with IBD have significantly more neutrophilic markers of N1 and fewer N2 markers than healthy individuals. Therefore, the ability of MSC-EXO to impede ERK signal transmission and ERK phosphorylation of neutrophils and promote neutrophil polarization towards the N2 phenotype greatly contributes to alleviating IBD (72). The high expression of miR-378a-5p in HucMSC-EXO results in decreased expression of NLRP3 inflammatory bodies, increased cell survival rate, and abrogates cell pyroptosis, alleviating DSS-induced IBD (73). A study found that Periodontitis and IBD, two types of inflammation, interact with each other. Periodontitis aggravates IBD and MSC-EXO produced by 3D culture promotes the expression of miR-1246, inhibits Nfat5, restores Th17 cell/Treg balance, and relieves the periodontitis of IBD patients (1). In addition, a study by Ting Zhang et al. found that M6A mRNA modification could maintain colonic epithelial cell homeostasis through NF-β-mediated anti-apoptotic pathways and that disruption of intestinal homeostasis is one of the possible triggers of IBD. Thus, m6A modification of mRNA could alleviate IBD by maintaining intestinal homeostasis and reducing apoptosis in intestinal epithelial cells (74). Although this study does not discuss whether MSCs and MSC-EXO contribute to the regulation of m6A modification of mRNA, it has been shown that MSCs and MSC-EXO promote m6A mRNA modification (56, 75). Regardless, more studies are needed to further explore whether MSCs and MSC-EXO can alleviate IBD by regulating m6A mRNA modifications. In summary, although there is evidence that MSCs and MSC-EXO can influence the development of IBD by regulating epigenetic modifications, their relationship, specific mechanisms of action, and pathways still need to be further explored. The regulation of immune cells by MSC in the inflamed gut is summarized in **Figure 3**.

### 3.2 Osteoarthritis

Osteoarthritis (OA) is considered to be a chronic inflammatory disease characterized by joint deformation and progressive degeneration of cartilage caused by an imbalance between osteogenesis and bone resorption activities, affecting approximately 15% of the world’s population, and accounting for approximately 10% of men and 18% of women over the age of 60. The incidence of OA will further increase with factors such as population aging and obesity rates, placing enormous economic pressure on society and people (76). OA not only damages the knee joint but also the spine, hand, and jaw joints, and is a major cause of disability in the elderly today (77). The development of OA is the result of a combination of environmental factors, genetic factors, and non-genetic factors such as being overweight. The main aim of treating OA is to reduce pain and improve movement. There are various strategies for the treatment of OA, which include options such as exercise, weight control, and other lifestyle treatments, as well as the use of drugs such as Duloxetine, Chondroitin, or Glucosamine. However, all of these treatments have their drawbacks, and when applied clinically the results vary considerably between individuals, do not cure OA, and are prone to relapse (78, 79). Therefore, there is a need to explore new treatment options.

MSCs have been used in recent years for the treatment of OA because of their potential to differentiate into chondrocytes and their ability to produce extracellular matrix. Tofiño-Vian M et al. found that adipose MSC-EXOreduces the expression of inflammatory mediators, particularly IL-6 and prostaglandin E2, to exert anti-inflammatory and protective effects on chondrocytes and suppress inflammation in OA. It has been suggested that chondrocyte destruction and loss of regenerative capacity are important factors in OA, and therefore promoting chondrocyte regeneration plays a very important role in the treatment of OA (80). Yubao Liu et al. found that MSC exosomal lncRNA KLF3-AS1 increases the expression of G-protein-coupled receptor kinase interacting protein-1 (GIT1) by sponging miR-206, promoting the regeneration of chondrocytes in OA through the lncRNA-KLF3-AS1/miR-206/GIT1 axis and inhibiting apoptosis to prevent and treat OA (81, 82). MSC-EXO is therefore considered a potential new cell-free therapeutic approach for the treatment of OA.

Epigenetic alterations are closely associated with aging and are contributing factors to aging-related diseases such as OA. Thus, age-dependent epigenetic regulation of gene expression plays a very important role in the development and progression of OA. The epigenetics of the growth factor BPM-7 (bone morphogenetic proteins 7), which is important for human articular chondrocyte activity, has been found to change with age, and promoter methylation of BPM-7 increases with age. The hypermethylation of the promoter of BPM-7 leads to abnormal bone formation and degradation eventually leading to OA (9, 83).

It was found that MSC-EXO could treat OA by modulating the DNA methylation of target molecules. The expression levels of NF-κB and associated coiled-coil-containing protein kinase (ROCK) are direct targets of miR-124 and miR-143 and were reduced in the MSC-EXO treatment groups, where the clinical manifestations associated with OA in mice were alleviated. NF-κB and ROCK are both implicated in the development of OA, as the activation of ROCK causes degeneration of cartilage, reduces bone production, and inhibits osteoblast degeneration (84) while NF-κB affects the expression of certain apoptosis regulators such as C-caspase3, Cyto-c, and Bax induces apoptosis and promotes the production of ADAMTs-5 and MMP-13, all of which contribute to the pathogenesis of OA (85). The expression of miR-124 is significantly reduced in IL-1β-induced arthritic mice, whereas the expression of NF-κB and ROCK is increased. MSC-EXO affects miR-124/NF-kB and miR-143/ROCK1/TLR9 signaling pathways by regulating the DNA methylation of the miR-124 promoter, which inhibits the expression of NF-kB and ROCK1, thereby affecting the onset and development of OA (86). It was found that MSC-EXO up-regulates the expression of miR-92a-3p, enhances chondrocyte formation, and inhibits chondrocyte degradation by targeting WNT5A. miR-92a-3p plays an important role in cartilage formation and degradation, and the expression of miR-92a-3p in OA cartilage is significantly lower than that in normal cartilage (87, 88). In addition, miR-92a-3p is an important regulator of Aggrecanase-1 (ADAMTS-4) and aggrecanase-2 (ADAMTS-5), and ADAMTS-4/5 plays a very important role in the development of OA (89). MSC-EXO affects the development of OA by up-regulating the expression of miR-92a-3p (87).In addition, the expression of circRNA_0001236 in OA cartilage is significantly lower than that in normal cartilage and is associated with the incidence of OA. The expression of circRNA_0001236 in chondrogenic MSC-EXO is significantly higher than that in undifferentiated MSC-EXO. MSC-EXO alleviates OA by up-regulating circRNA_0001236 expression in chondrocytes (90). Infrapatellar fat pad (IPFP) MSC-derived exosomes (MSCIPFP-Exos) inhibits the expression level of miR-100-5p and the binding of mTOR to miR-100-5p. MSCIPFP-Exos reduces the autophagy level of chondrocytes and improves the severity of OA *in vivo* by inhibiting the mTOR autophagy pathway (91). It is demonstrated that TGF-β1-stimulated BMSC-derived exosomes (BMSCs-ExoTGF-β1) are highly expressed with miR-135b, which promotes M2 polarization of macrophages, inhibits M1-polarization, and repairs cartilage damage (92). All the above studies suggest that MSC-EXO can alleviate the occurrence and development of OA by regulating epigenetic modification

Interestingly, it has been shown that certain molecules can influence the differentiation of MSCs and the secretion of MSC-EXO through their own epigenetic modifications to affect OA. For example, microRNAs are involved in the subtle control of gene expression in osteoblasts/chondrocytes, promoting MSCs toward osteogenesis. A study by Feng Liu et al. found that the knockdown of the lncRNA CIR gene results in significantly higher expression of the chondrogenic markers SOX9, Aggrecan, and COL2A1 than controls, demonstrating that the presence of lncRNA CIR inhibits the differentiation of hUC-MSCs into chondrocytes and that there is a relationship between lncRNA CIR and the development of OA. Further studies revealed that lncRNA CIR promotes EZH2-mediated methylation of the ATOH8 promoter, which inhibits the expression of ATOH8, thereby suppressing the differentiation of hUC-MSCs towards chondrocytes (93). In summary, although the specific mechanisms and molecular pathways between epigenetic modifications and MSCs still need further exploration and validation, it is undeniable that there is a link between them, and this link may be a breakthrough point in the treatment of inflammatory diseases such as OA.

### 3.3 Systemic lupus erythematosus

Systemic lupus erythematosus (SLE) is a chronic inflammatory disease involving multiple systems and organs, mediated by the autoimmune system, with varying degrees of presentation, course, and prognosis, ranging from mild skin and mucosal tissue damage to severe damage to multiple organs and the central nervous system. Environmental factors play a role in the pathogenesis of SLE (94). Defects in the early complement components C1q, C1r, C1s, C4, C2, and TREX1 are high-risk factors associated with the development and progression of SLE. Although the exact gene has not been identified, the risk of SLE is 10 times higher in women than in men, and the risk in women with Klinefelter syndrome (47, XXY) is 14 times higher than in men, which is sufficient evidence to link the development of SLE to genes located on chromosome X (95). Severe and persistent inflammation in SLE causes damage to a variety of organs, which is one of the causes of SLE complications. Complications associated with SLE include atherosclerosis, neurological defects, and kidney disease. About half of all SLE patients develop nephritis, which is a major cause of death in SLE patients (96). Studies have found that autoantibodies often appear before the clinical manifestations of SLE when they cause damage to the immune system and normal physiological functions, so early diagnosis and treatment can help improve the remission rate and prognosis of SLE patients. Immunomodulators and immunosuppressants are used to improve the immune system and treat SLE to maintain minimum activity (97). Since steroids can place a significant burden on the body and lead to sequelae of events such as cardiovascular disease, B-cell depletion (BCD) without any or minimal use of steroids is also a possible treatment for SLE therapy (98, 99).

MSC-EXO has also been used in studies of SLE treatment due to their multidirectional differentiation and less immunogenic properties, MSC-EXO can maintain homeostasis of M2-type macrophages by promoting polarization of macrophages towards an anti-inflammatory phenotype and activation of Treg cells. Inhibiting the activation of effector cells involved in the innate and adaptive immune response suppresses the immune response and alleviates the autoimmune response induced by autoantibodies in SLE (100). Reduced expression of CD4, CD25, and FOXP3 is a major genetic defect in SLE and MSCs can promote CD4+, CD25+, and FOXP3+ inducible Treg cells in SLE peripheral blood mononuclear cells (PBMCs) by releasing Transforming Growth Factor Beta 1 (TGFβ1) (101). Jang E et al. showed that human bone marrow (hBM)-MSCs administration in a mouse model of lupus nephritis relieves the condition by inhibiting the development of T follicular helper cells (Tfh)cells and the subsequent activation of humoral immune components, decreasing autoantibody levels and the incidence of proteinuria in mice, and improving survival (102). Clinical trials have also shown the role of MSCs in the treatment of SLE, with results from randomized controlled trials showing some relief of symptoms in the MSCs-treated group, a significant reduction in disease activity index and 24h proteinuria, and an increase in complement C3 (103). Although the results of the study show that MSCs are effective in the treatment of SLE, their efficacy is still low compared to other diseases, and therefore further research is still needed to verify whether MSCs and MSC-EXO can be used in the clinical treatment of SLE patients.

Epigenetic modifications such as abnormal DNA methylation and histone modifications play a key role in the pathogenesis of SLE, and the main epigenetic alteration in SLE patients is the overall hypomethylation of CD4+ T cells. Patients with active SLE show an overall downregulated expression level of H3 and H4 acetylation in CD4+ T cells *in vivo*, which leads to an increased expression of autoimmune-related genes and an elevated risk of developing autoimmune diseases. At the miRNA level, upregulation of miR-148a and miR-21 in the MRL/LPR mouse model results in reduced DNA methyltransferase 1 (DNMT1) expression and decreased DNA methylation levels, exacerbating lupus (104).In SLE patients, the expression level of mRNA methyltransferase NSUN2 in CD4+T cells is decreased, and the Methylation level of mRNA m5 C Methylation in CD4+T cells is abnormally increased. Epigenetic defects, especially the abnormal expression of cytokines and co-receptors caused by abnormal DNA methylation and histone modification, are also considered to be one of the important causes of non-SLE immune activation and tissue damage (105, 106). Therefore, correcting the aberrant epigenetic modifications in SLE patients is a potential therapeutic modality for SLE.

MSC-EXO can relieve SLE by modulating epigenetic modifications. For example, MSC-EXO was found to improve the reduction of bone mass in lupus and alleviate the symptoms of SLE. Fas-deficient-MRL/LPR mice lack FAS, resulting in the failure of miR-29b release, leading to a high level of miR-29b in the cell. Moreover, the deficiency of FAS results in the decreased expression of DNMT1 in BM-MSCs of MRL/LPR mice, resulting in hypomethylation of the notch1 promoter and activation of NOTCH signaling, which in turn impairs osteogenic differentiation. However, MSC-EXO reduces the levels of miR-29b and upregulates DNMT1 in recipient mouse cells, thereby restoring DNMT1-mediated methylation of the NOTCH1 promoter and downregulating the expression of NOTCH1 and NICD, rescuing the function of BM-MSCs in MRL/LPR mice to some extent. This implies that MSC-EXO could rescue the BM-MSC of MRL/LPR mice by promoting the release of miR-29b and thus regulating the miR-29b/DNMT1/Notch epigenetic cascade to treat SLE to a certain extent (107). In addition, the aging of MSCs is closely related to the incidence of SLE. MiR-146-a can be targeted TRAF6/NF-KB signaling pathway to participate in the aging of MSCs, as a study found that exosomes derived from SLE serum have significantly decreased expression of miR-146-a, along with increased SA- KB-gal positive cells. This is likely linked to MSCS aging mechanisms in SLE patients, where epigenetic modification affects the occurrence and progression of the disease by affecting MSCs (108). However, studies on the treatment of SLE by MSCs and MSC-EXO through the regulation of epigenetic modification are largely lacking. More studies are still needed to verify the idea that MSCs can play a therapeutic role in SLE through epigenetic modification.

### 3.4 Other diseases

In addition to the above-mentioned inflammatory diseases, MSCs, and MSC-EXO can also act on other inflammatory and non-inflammatory diseases through the regulation of epigenetic modifications. For instance, in acute liver injury, MSC-EXO induced expression of SLC7A11 protein causes an increase in CD44 and OTUB1, which mediates deubiquitination and removes aberrant ubiquitination of SLC7A11, thereby increasing the stability of SLC7A11, activating system XC-, and preventing CCL4-induced hepatic cytophosis. This provides a new idea for the prevention and treatment of acute liver injury caused by cytophosis (109). Another study found that hUC-MSC-EXO promotes miR-4553p expression when subjected to IL-6 stimulation. Western-blot and QRT-PCR analyses showed that PIK3r1 expression is significantly reduced in the presence of miR-4553p at both the macrophage mRNA and protein levels. PI3K is a key factor in inhibiting the activation of IL-6-related signaling pathways, suggesting that miR-4553p inhibits macrophage activation by suppressing the expression of the target gene PIK3r1. In effect, hUC-MSC-EXO inhibits the release of IL6, as well as other inflammatory factors from macrophages by promoting the expression of miR-4553p.Targeting PIK3r1. suppresses the over-activation of immune cells such as macrophages/monocytes, reducing inflammation, ameliorating liver damage, and maintaining systemic homeostasis (110).

Studies have found that lncGm36569 is increased in ASCI mouse models and anoxic cell models treated with MSC-EXO. Bioinformatics analysis and luciferase analysis showed that lncGm36569, as a competitive RNA of miR-5627-5p, induced upregulation of FSP1. The overexpression of miR-5627-5p inhibits the therapeutic effect of lncGm36569 in the treatment of neuronal iron osteoporosis. Therefore, MSC-EXO can inhibit siderosis in neurons by regulating the expression of lncGm36569 and acting on miR-5627-5p/FSP1 axis (111). Nan Zhang et al. found that MSC-EXO could deliver non-coding developmental regulatory RNA (FENDRR) to tissues and cells, FENDRR can also regulate the expression of TEA domain transcription factor 1(TEAD1) by binding with miR-28. Moreover, it was found that when miR-28 was inhibited, atherosclerotic plaques were reduced. The deletion of TEAD1 reduces the inhibition of miR-28 and aggravates AS. Therefore, it indicates that MSC-EXO competitively binds to miR-28 with TEAD1 by secreting FENDRR, and alleviates the occurrence and development of AS (112). Yuli Zhang et al. found that imiquimod (IMQ) induces epidermal proliferation in mice. hucMSC-EXO inhibits IMQ-induced phosphorylation of signal sensors and transcriptional 3 (STAT3) activators in mIce skin and human keratinocyte (HaCaT) cells in addition to reducing the expression of IL-17. Thus alleviating psoriasis-like skin inflammation in mice. Therefore hucMSC-EXO may be an effective treatment for psoriasis (113). Ge Gao et al. found that exosomal circular RNA (circ_0006790), which was carried by MSC-EXO, regulated DNA methylation of S100A11 and inhibited transcription of S100A11 by binding with CBX7 and recruiting methyltransferase to the promoter region. Blocking the immune escape of adenocarcinoma cells is a new prospect for pancreatic ductal adenocarcinoma (PDAC) treatment (114). The role of MSC-EXO in epigenetics is summarized in **Table 3**.

**Table 3:** Effects of MSC-EXO on epigenetics.

Disease	Study model	Function	Reference
Inflammatory bowel disease	*In vivo* model/mice	Inhibits the phosphorylation of IκBα	(70)
Inflammatory bowel disease	*In vivo* model/BABL/C mice *In vitro* model/FHC	Inhibits ERK phosphorylation	(72)
Inflammatory bowel disease	*In vivo* model/C57BL/6J mice	Decreases the expression of miR-1246	(10, 73)
Osteoarthritis	*In vivo* model/mice *In vitro* model/Primary chondrocytes	Regulates DNA methylation of miR-124 promoter	(86)
Osteoarthritis	A sample of articular cartilage from a patient with OA knee joint. Normal cartilage samples were taken from patients with no history of OA or rheumatoid arthritis	Upregulates the expression of miR-92a-3p	(87–89)
Osteoarthritis	Human clinical samples	Upregulates the expression of circRNA-0001236	(91)
Osteoarthritis	*In vivo* model/SD rats at 12 weeks old *In vitro* model/BMSC	Inhibits the expression of miR-100-5P	(92)
Systemic lupus erythematosus	*In vivo* model/C3MRL-Faslpr/J(MRL/lpr) *In vitro* model/BMSC	Reduces the levels of miR-29b	(107)
Acute liver injury	*In vivo* model/C57BL/6 J mice	Mediates SAC7A11 deubiquitination	(109)
Liver damage	*In vivo* model/C57BL/6 mice *In vitro* model/THP-1	Promotes the expression of miR-4553p	(110)
Acute spinal cord injury	*In vivo* model/C57BL/6 mice *In vitro* model/HT-22 and HEK-293 T	Promotes the expression of lncGm36569	(111)
Atherosclerosis	Clinical samples *In vivo* model/ApoE−/− mice *In vitro* model/HUV-EC-C	Triggers the release of lncRNA and regulates TEAD1 expression	(112)
Psoriasis	*In vivo* model/C57BL/6 mice *In vivo* model/HaCaTcells	Inhibits the phosphorylation of STAT3	(113)
Pancreatic ductal adenocarcinoma	*In vivo* model/BALB/c mice *In vivo* model/PANC-1(CL-0184)and CFPAC-1(CL-0059)	Regulates the DNA methylation of S100A11	(114)

## 4 Conclusion and prospects

Epigenetic modification is a very complex mechanism for regulating gene expression without altering DNA sequence, and the interrelationship between metabolic alterations and epigenetic remodeling is one of the hallmarks of cancer and a causal factor in the pathogenesis of many diseases (115). The study of epigenetic modification may provide new ideas for the treatment of certain refractory diseases through the regulation of DNA methylation, m6A modifications, and other epigenetic modifications by MSCs and MSC-EXO. It also provides new insights into the study of the mechanisms of MSCs and MSC-EXO for the treatment of inflammatory diseases. Although epigenetic modifications include DNA, non-coding RNA, and histone-related modifications, few studies have examined the role of MSCs and MSC-EXO through modulation of histone modifications, and most studies have focused on DNA methylation modifications and non-coding RNA modifications. However, studies have shown that histone modifications can also affect inflammatory diseases through certain pathways. For example, the TLR signaling adapter BCAP can regulate the conversion of inflammatory reparative macrophages by promoting histone lactation modifications. Moreover, lactate and histone modifications lead to the expression of tissue repair genes, restoring the tissue repair properties of macrophages, as mice recover from DSS-induced enteritis (11). Impaired histone modification of T cells and CpG DNA methylation in SLE increases the expression of IL-17a, breaks the balance between cytokines, and aggravates the pathological damage of SLE, exacerbating the pathological damage in SLE (116). In addition, the influence of histone lactate modification on diseases is bidirectional. Studies have found that under the condition of LPS-induced inflammation, glycolysis activity is enhanced, lactic acid production is increased, and histone lactate modification is significantly up-regulated, promoting the occurrence and development of inflammation. However, in the late stage of M1 macrophage polarization, the increase of histone lactate modification promotes the expression of homeostasis genes involved in the damage repair process (32). Whether and how MSC-EXO promotes histone modification towards alleviating inflammation by regulating the bidirectional effect of histone modification remains to be further investigated. Although many studies have shown that MSC-EXO can alleviate inflammatory diseases by regulating epigenetic modifications, there are still fewer papers on the specific signaling pathway through which MSC-EXO affects epigenetic modifications. The discovery of specific upstream or downstream signaling pathways through which MSC-EXO affects epigenetic modification to relieve IBD will likely provide a more promising foundation for the development of relevant therapeutics for IBD and other inflammatory diseases. Further research exploring the relationship between epigenetic modification and MSC-EXO is necessary, and disease pathogenesis and therapeutic research will bring great advances.

At present, there are studies on MSCs and epigenetics, most of which are about the effect of epigenetic modification on MSCs differentiation, while there are few related contents about how MSCs regulate epigenetic modification. There are also studies on MSC-EXO alleviating inflammatory diseases by regulating epigenetic modification, but most of such studies focused on the MSC-EXO effect through modulating certain molecules and mRNA expression and not directly regulating epigenetic modification. Although studies have shown that MSC-EXO can affect the DNA methylation of promoters, more studies are needed to prove the reliability of this conclusion. As a cell-free therapy, MSC-EXO is superior to MSC in treatment because it is more stable and could reduce the inherent safety risks associated with the administration of cell-based therapy, including the risk of occlusion in the microvasculature, as well as possible immune recognition by the host system. Moreover, MSC-EXO possesses enhanced delivery of exogenous biological particles to the target site and directly into the cytosol, circumventing the lysosomal-endosomal pathway, and consequently elevating transfection efficiency (117). As a result of their small sizes and other camouflage strategies, exosomes are capable of evading the mononuclear phagocytic system’s clearance, leading to extended circulatory time for passive targeting of inflammatory and cancerous cells (15). These special properties among others give MSC-EXO enormous potential over the parental cell therapy in regenerative medicine and cancer therapy. Considering the crucial role of epigenetic modification in the occurrence and development of inflammatory diseases and the efficient anti-inflammatory effect of MSC-EXO, the application of MSC-EXO to regulate epigenetic modification may be a potential therapy for inflammatory diseases.

## Author contributions

Conceptualization, ZZ, LZ, and BW; funding acquisition, DO; project administration, FM; software, DO; visualization, FM; writing—original draft, ZZ, LZ, and BW; writing—review & editing, DO. All authors contributed to the article and approved the submitted version.
